# Supplementary material for: Are carbon emissions trading and green financial instruments synergistic? -Comprehensive quantitative research based on content analysis
Source: PLoS One. 2024 Mar 7;19(3):e0298601. doi: 10.1371/journal.pone.0298601 (PMC10919601; doi:10.1371/journal.pone.0298601)
Supplement: S1 Data — (ZIP) [file pone.0298601.s001.zip › Data/地级市Z_F工作报告.zip!/▒▒╛⌐/└┤╫╘╣1⁄2╓┌║┼í╛┬φ┐╦╩2╛▌═°í┐_2009.docx]

　　——2009年1月12日在北京市第十三届人民代表大会第二次会议上

　　北京市市长 郭金龙

各位代表：

　　现在，我代表北京市人民政府，向大会报告政府工作，请予审议，并请市政协各位委员提出意见。

　　一、过去一年工作回顾

　　2008年，是首都发展史上具有特殊重要意义的一年。在党中央、国务院和中共北京市委的领导下，全市人民认真贯彻党的十七大精神，深入贯彻落实科学发展观，紧紧围绕“营造良好局面、办好一件大事”，团结奋斗，扎实工作，无私奉献，较好完成了市十三届人大一次会议确定的各项任务，胜利实现了“新北京、新奥运”战略构想，首都改革开放和现代化建设迈入新的发展阶段。

　　(一)北京奥运会、残奥会筹办工作圆满完成。

　　坚决贯彻落实中央指示精神，全面实践“绿色奥运、科技奥运、人文奥运”理念，坚持节俭办奥运、廉洁办奥运，创新筹办体制机制，促进职能整合与工作融合，实现了办一届有特色、高水平奥运会、残奥会的目标，做到了让国际社会满意、让各国运动员满意、让人民群众满意，为实现中华民族的百年梦想作出了重要贡献。

　　奥运建设取得新成绩。经过七年多艰苦努力，高质量建成奥运场馆及相关设施，“鸟巢”、“水立方”等成为时代精品，谱写了中国建筑史的光辉一页。地铁10号线一期、奥运支线、机场线开通运营，轨道交通运营里程达到200公里。机场二高速、京平高速等建成通车，高速公路通达每个区县。首都机场T3航站楼、京津城际客运专线和新北京南站投入运营。南水北调北京段主体工程完工，实现冀水进京。完成“0811”电力强网工程，初步构建起一流的城市电网。太阳宫、郑常庄燃气热电厂和草桥供热厂建成投产，新增集中供热能力2680万平方米。城市基础设施服务保障水平进一步提高。

　　城市运行井井有条。水电气热等市政设施运转良好，邮政通信和信息网络通畅安全。严格落实“双进入”体制，场馆运行内外保障无缝衔接、实现赛时“零故障”，成功实施奥运人工消雨，确保了开闭幕式和各项赛事平稳有序进行。认真组织实施机动车单双号限行措施，赛事交通与城市交通和谐运转，全年公共交通出行比例达到36.8%。全力保障鲜活农产品供应，消费市场繁荣稳定。全面落实“三案一室”，搭建指挥监测平台，城市运行和应急管理明显加强。

　　城乡环境明显改善。严格落实奥运空气质量保障措施，赛会期间空气质量天天达标，全年二级以上天数达到74.9%。奥林匹克公园中心区交通实现“零排放”，奥运场馆生活垃圾全部分类收集、集中处理。城乡生活垃圾无害化处理水平继续提高，市区和郊区污水处理率分别达到93%和48%，再生水利用率达到57%。高度重视生态建设，完成山区绿化5267公顷，新增城市绿地518公顷，人均公共绿地达到13.6平方米。扎实推进城乡环境建设，60个城市“边角地”、102个城乡结合部行政村、117条主要大街、一批胡同及老旧小区整治取得明显成效，营造了干净整洁、优美靓丽的城市环境。

　　服务保障优质高效。奥运外事服务周到细致，机场抵离方便快捷。奥运村、残奥村实现“安全运行零事故、各项服务零投诉、礼宾活动零失误”，奥运大家庭饭店、签约饭店服务接待得到广泛好评。全面实施《奥运食品安全行动纲要》，强化从农田到餐桌全程监控，涉奥餐饮万无一失。加强公共卫生监控、预警和应急救援，高标准做好奥运医疗卫生保障工作，全市没有发生重大突发公共卫生事件。窗口行业服务优质文明，旅游接待热情规范。高水平完成无障碍设施建设，组织40万残疾人员参与残奥会活动，为残疾人朋友提供了体贴入微的服务，尊重关爱残疾人的社会氛围更加浓厚。

　　“平安奥运”全面实现。安保工作组织严密、措施到位，广大市民积极参与维护社会治安，未发生暴力恐怖事件、危害国家安全和社会稳定的重大政治事件以及大规模群体性事件。深入开展安全生产隐患排查治理，扎实推进百日督查专项行动，强化高危行业企业安全监管，安全生产事故数量和死亡人数为近年最低。健全食品安全监管、检测和信用体系，严格执行药品流通全过程的准入和监管标准，群众食品药品安全得到有力保障。制定《突发事件应对法》实施办法，完善各级各类应急预案，组织开展综合与专项演练，加强公共安全风险控制，全年无重大突发公共事件。

　　社会氛围文明和谐。深入开展“迎奥运、讲文明、树新风”活动，文明出行、排队礼让蔚然成风，市民文明素质和城市文明程度明显提高。170万志愿者参与奥运服务，创历届奥运会和我国志愿者服务之最，为北京树立起全新的人文坐标。认真实施奥运教育计划，积极组织后备观众，赛场氛围友好热烈。高水平做好新闻宣传和媒体服务工作，营造了开放向上的奥运舆论环境。广泛开展各种文化活动，成功举办第六届奥林匹克文化节，文化广场演出、奥林匹克公园公共区文化活动丰富多彩，展现了中华文化魅力，促进了中外文化交流。

　　(二)经济社会保持又好又快发展。

　　坚决贯彻中央决策部署，坚持“两手抓、两不误、两促进”，保持了经济发展、社会祥和的良好局面，发展成果惠及人民群众。

　　首都经济较快发展。初步预计，地区生产总值突破1万亿元，比2007年增长9%，人均地区生产总值超过8000美元。居民消费价格指数为105.1%，低于全国平均水平。地方财政收入达到1837.3亿元，增长23.1%。全社会固定资产投资完成3900亿元左右，接近2007年水平；社会消费品零售额超过4500亿元，增长20%以上，消费对经济增长的拉动作用显著增强。制定实施促进首都金融业、旅游业发展意见，加快高端产业功能区建设，现代服务业较快发展，第三产业比重达到73%左右。首钢顺利压产400万吨，化工二厂、有机化工厂全面停产，“五小”企业加快退出，各类建筑节能改造超额完成任务，万元地区生产总值能耗、水耗和化学需氧量、二氧化硫排放量等调控目标全面实现。首都经济继续保持“增长较快、结构优化、效益提高”的良好势头。

　　新农村建设扎实推进。市政府“三农”投入171.2亿元，比2007年增加40.6亿元。全面落实和强化各项惠农政策，大幅提高种粮补贴标准，扩大生态林补偿机制覆盖范围，绿化隔离地区政策进一步完善。行政村配备大学生“村官”目标基本实现。编制完成了400个村庄规划，加强农村基础设施和公共服务设施建设，公共交通、信息网络基本实现村村通，农民安全饮水目标提前两年全面实现，平原地区村庄生活垃圾实现密闭化管理。制定集体林权制度改革意见，规范发展农民专业合作社，农村集体经济产权制度改革步伐加快。开展村镇银行、农村资金互助社和小额贷款公司试点，政策性农业保险参保农户达到16.4万户。推进现代农业服务体系建设，拓宽农村科技服务渠道，动植物疫病防控、兽医管理、农资市场监管得到加强，设施农业、观光农业、民俗旅游等取得长足进步。制定实施山区协调发展总体规划，区县合作机制初步建立，新一轮山区农户搬迁顺利进行，小流域综合治理、废弃矿山生态修复取得新成绩，生态涵养发展区建设步伐加快。

　　社会事业繁荣发展。市级财政对教育科技、医疗卫生、文化体育的投入达到337.6亿元，增长19.2%。扩大“两免一补”政策范围，免收义务教育阶段城市户籍学生杂费、农村地区非农户籍学生教科书费。完成100所小学标准化建设任务，改善了140所农村寄宿制学校生活条件，提高了山区中小学教师工资。基本建立起多层次的教育资助体系，对24万名困难家庭学生给予资助，外来务工人员子女接受义务教育工作继续加强。发布实施中长期科学和技术发展规划纲要，“科技奥运”取得一批重大创新成果，全市专利申请数量、技术交易额保持较快增长。社区卫生服务机构标准化建设和“收支两条线”改革全面完成。投入2.8亿元，为187个乡镇和1391个村的卫生服务机构配备标准化设备。推出了一批方便群众看病就医的新措施，政府出资为农民统一购买村级医疗卫生服务，解决了乡村医生基本待遇和养老保障问题。深化文化体制改革，推进电影公司等转企改制，组建北京演艺集团，积极培育文化品牌。加强农村、社区文化建设，建成一批公共文化服务设施，首批33家博物馆、纪念馆免费向社会开放，“周末场演出计划”和“文艺演出星火工程”顺利实施。完成了国家非物质文化遗产名录申报，广播电视、新闻出版、文学艺术和哲学社会科学繁荣发展。行政村全民健身设施建设全面完成，各类群众体育活动广泛开展，北京运动健儿勇夺15枚奥运会奖牌、19枚残奥会奖牌。

　　人民生活持续改善。城镇居民人均可支配收入达到24725元，农民人均纯收入10747元，分别实际增长7%和6.5%。大力开发就业岗位，城镇新增就业42万人，登记失业率为1.82%。健全就业援助制度，帮扶10.1万名就业困难人员实现就业、10.5万名农村劳动力转移就业，城乡“零就业家庭”基本消除。推行农民工实名制管理，建立建筑业农民工工资保障金、劳务费和工资专用账户制度，及时解决拖欠工资问题。为城乡60岁以上无社会保障居民发放养老补贴，新型农村养老保险、城镇无业居民大病医疗保险制度全面推行，城乡养老、医疗保障制度基本实现全覆盖。落实医疗保险“一降三升”惠民政策，完善“一老一小”大病医疗保险制度，提高新农合门诊报销水平，有效减轻了群众医疗费用负担。制定农村低保最低标准，提高城镇居民社会保障待遇水平，在全国率先启动了社会保障卡工程。实施老年人优待和养老服务补贴制度，社会福利、优抚安置、慈善事业、红十字工作取得新进展。800万平方米廉租房、经济适用房和限价商品房建设任务全面落实，完成4049户、12.1万平方米城镇危房解危工作，为1279户农村困难家庭翻修住房。积极探索风貌保护新模式，有序推进历史文化保护区整治，修缮旧城胡同44条、院落1954个。发布社会建设实施纲要，广泛开展和谐社区、和谐村镇、和谐社会组织创建活动，社会建设迈出新步伐。深入推进矛盾纠纷排查化解工作，开展大接访和拉网式排查，实施重信重访专项治理，化解信访积案。经过各区县、各部门、各单位的共同努力，59件重要实事全部落实，“五无”目标全面实现。

　　改革开放取得新进展。制定了深化行政管理体制改革的实施意见和市政府机构改革方案，事业单位改革和政府购买公共服务试点稳步推进。市与区县分税制财政管理体制改革迈出重大步伐，促进区县财力增长和事权与财权相匹配。加快“调改剥退”步伐，积极推进国有资本布局调整和国有企业改制重组，国有资本收支预算和国有资产评估管理不断规范。设立市级中小企业创业投资引导基金，成立中小企业信用再担保公司，投融资平台和要素市场建设取得新突破。继续推动中关村做优做强，以企业为主体的创新体系不断完善。积极推动“双自主”产品开拓国际市场，大力发展服务外包，地区进出口总额保持快速增长。创业投资和环保、医药领域利用外资取得重大进展，实际利用外资超过60亿美元，增长20%以上。圆满完成亚欧首脑会议服务保障工作，科博会、文博会、京港洽谈会等经贸活动取得新成效。深化“四个服务”，为中央单位和驻京部队办理重要服务事项390件。

　　民主法制建设得到加强。市人民政府及组成部门全面执行市人民代表大会及其常委会的决议，坚持做好向市人大常委会报告专项工作，自觉接受人大监督。认真听取各民主党派、工商联、无党派人士和人民团体的意见，积极参加市政协常委会、议政会等会议。共办理全国和市人大代表议案、建议1517件，政协常委会建议案、政协提案1175件。推进政府信息公开，完善了重大决策的规则和程序，实行公示和听证制度，强化审计监督。健全基层群众自治制度，深入推行村务、厂务公开。加强政府法制建设，共提请市人大常委会审议地方性法规5项，制定、修订政府规章8项。奥运立法和相关法规的有效实施，为奥运会成功举办创造了良好的法制环境。扎实开展普法宣传教育活动，推进公益法律服务体系建设。全面落实民族宗教政策，切实维护归侨侨眷合法权益，开展多领域对台交流与合作。依法落实计划生育奖励和优惠政策，稳定低生育水平。妇女、儿童、老龄事业健康发展。国防后备力量建设得到加强，双拥共建活动深入开展，“连连通油路”全面实现。驻京人民解放军和武警部队广大官兵为奥运筹办、为首都发展作出了重要贡献。

　　各位代表！

　　在奥运筹办的关键阶段，面对突如其来的南方部分地区严重低温雨雪冰冻灾害和汶川特大地震，首都人民积极响应党中央、国务院的号召，第一时间行动起来，广泛开展支援救灾工作，社会各界踊跃捐款捐物23.63亿元。大批消防官兵、医务工作者、各领域专家、志愿者奔赴地震灾区，参与抗震抢险和救治伤员。全市上下密切配合，妥善安置灾区来京伤员和学生，圆满完成6.66万套过渡安置房援建任务。扎实推进对口支援什邡市灾后恢复重建工作，认真落实援建工作方案和总体框架协议，累计拨付资金24.98亿元，各项援建工程进展顺利，对口支援取得重要阶段性成果。

　　2008年10月以来，面对国际国内经济形势急剧变化的严峻挑战，我们坚决贯彻落实中央决策部署，迅速制定实施了保持首都经济平稳较快发展的29条具体措施，计划今明两年安排市区两级政府投资1400亿元左右，预计带动社会投资10000亿元。研究部署了扩大内需的若干专项工作，出台了提高社会保险待遇标准、降低社会保险缴费率等多项政策，为“保增长、促民生”提供了有力保障。

　　各位代表！

　　通过一年来的实践，我们更加深刻地体会到，做好首都各项工作，必须深入贯彻落实科学发展观，坚决贯彻中央的大政方针和对北京市工作的指示精神，不断深化对首都经济社会发展规律和特点的认识，紧密结合实际，创造性地开展工作。同时，我们也获得了新的启迪：

　　第一，必须继承奥运财富，发展奥运成果。奥运筹办是科学发展观的成功实践，留下了弥足珍贵的物质精神财富，为首都长久繁荣发展提供了强大动力。我们一定要认真总结、继承巩固奥运筹办经验，倍加珍惜、大力弘扬奥运精神。特别是要继续坚持奥运三大理念，加快建设“人文北京、科技北京、绿色北京”。

　　第二，必须增强首都意识，拓宽国际视野。北京充分享受了首都的发展优势，要更加全面地履行好“四个服务”职责，更好地落实各方面工作走在全国前列的要求。要强化“窗口”意识，树立全球视野和战略眼光，以更加积极的姿态走向世界，加快建设现代化国际城市。

　　第三，必须坚持以人为本，发挥市民主体作用。要时刻把人民群众的根本利益放在第一位，下大气力解决好人民群众最关心、最直接、最现实的利益问题，使发展成果更好地惠及人民群众。要尊重人民主体地位，发挥人民首创精神，着力完善政府服务管理、社会组织动员等体制机制，依靠人民群众推动科学发展、促进社会和谐。

　　第四，必须坚持统筹兼顾，增强处理利益关系的能力。要深入研究和科学认识首都现代化建设的各种重大关系，全面把握来自各方面的利益诉求，自觉运用统筹兼顾的根本方法，找准决策、政策和工作的平衡点，凝聚各方面的智慧与力量，发挥各方面的积极性与创造性，努力开创优势互补、互利共赢的发展局面。

　　第五，必须加快管理创新，提高政府效能。要按照建设服务政府的要求，加快行政管理体制改革，着力转变政府职能，进一步健全政府职责体系，强化社会管理与公共服务。要深入推进管理创新，建立健全部门间协调配合机制，加强基础工作，规范行政行为，降低行政成本，提高行政效能和服务水平。

　　我们也清醒地认识到，首都经济社会发展和政府工作中还存在不少问题，主要表现在：经济结构调整仍需进一步深入，自主创新能力有待进一步提高，发展方式需要进一步转变；城乡之间、区域之间、部分区县内部发展的差距比较大，统筹城市与农村、北城与南城、平原与山区发展，推动形成城乡经济社会发展一体化新格局需要下更大的力气；人口资源环境矛盾十分突出，实现首都可持续发展的任务相当艰巨；城市服务能力需要不断增强，管理体制机制有待继续创新；首都文化软实力和文化生产力有待进一步提升，将文化优势转化为现实生产力需要不断努力；在劳动就业、社会保障、收入分配、义务教育、医疗卫生、住房保障、食品药品和安全生产等方面，一些关系人民群众切身利益的问题需要加快解决；推进重点领域和关键环节的改革还需要加大力度，优化发展环境的任务依然很重；政府职能转变还不到位，行政效能和服务水平需要进一步提高，建设服务政府还有许多工作要做。

　　我们一定要保持清醒头脑，增强忧患意识，高度重视、不断解决发展中存在的突出矛盾与问题，更加努力地做好政府各项工作，决不辜负人大代表、政协委员的信任，决不辜负党和人民的期望！

　　各位代表！

　　过去一年的成就举世瞩目、来之不易，这是党中央、国务院坚强领导的结果，是全国人民大力支持、举国体制坚强保障的结果，凝聚了全市人民的智慧、心血和汗水。在这里，我代表市人民政府，向全市各族人民，向人大代表、政协委员，向各民主党派、各人民团体、社会各界人士，向中央各部门、兄弟省区市，向驻京解放军和武警部队官兵，向来自全国各地的劳动者，向广大奥运志愿者，向所有关心支持首都建设的港澳同胞、台湾同胞、海外侨胞和国际友人，表示崇高的敬意和衷心的感谢！

　　二、2009年工作的主要任务

　　2009年是新中国成立60周年，也是北京奥运会、残奥会成功举办后，首都发展进入新阶段的第一年。深入贯彻落实科学发展观，继承奥运财富，巩固奥运成果，健全城市可持续发展的长效机制，保持首都经济社会平稳较快发展，具有十分重要的意义。

　　当前，国际金融危机对首都经济的影响已经显现：房地产市场持续低迷，工业生产、外贸出口增幅下降，部分企业经营困难，地方财政收入增速下滑，经济增速回落。由于首都经济服务主导型和总部经济的特征，国际金融危机的影响还存在一定的滞后性，首都经济发展面临着诸多不确定因素。积极主动地打好应对危机与挑战这场硬仗，保持首都经济平稳较快发展，是2009年政府工作的首要任务，也是对我们新的重大考验。

　　与此同时，我们也要看到，首都经济社会发展具有很多有利条件。我国发展仍处于重要战略机遇期，中央出台了一系列扩大内需、促进经济发展的政策措施，为克服国际金融危机的冲击和影响提供了重要保障。奥运会成功举办，首都的发展环境明显改善，国际影响力、竞争力进一步提高，对人才、技术、资金等形成了新的吸引力。特别是首都经济处在结构升级、质量提升的关键阶段，科技创新、文化创意、综合服务的优势突出，拥有良好的发展条件和充沛的内在动力。我们要坚决贯彻落实中央决策部署，充分发挥首都优势，深化改革开放，积极应对国际经济环境的变化，变压力为动力，化挑战为机遇，统一思想、振奋精神、增强信心、埋头苦干，更加扎实地做好各项工作，向党和人民交上新的合格答卷！

　　2009年政府工作总的要求是：全面贯彻落实党的十七大和十七届二中全会、三中全会、中央经济工作会议精神，认真学习领会胡锦涛总书记在纪念党的十一届三中全会召开30周年大会上的重要讲话精神，深入贯彻落实科学发展观，总结运用奥运经验，大力弘扬奥运精神，坚定信心，抓住机遇，着力优化发展环境，着力扩大内需，着力推动自主创新，着力发展优势产业，着力深化改革开放，着力改善民生，保持首都经济平稳较快发展和社会祥和稳定，加快建设“人文北京、科技北京、绿色北京”，以建设繁荣、文明、和谐、宜居的首善之区的优异成绩，迎接新中国成立60周年。

　　综合考虑各方面因素，全市经济社会发展主要目标是：力争实现地区生产总值增长9%；城镇登记失业率控制在2.5%以内；城乡居民收入实际增长6%以上；居民消费价格指数保持在103.5%以内；地方财政收入增长10%以上；万元地区生产总值能耗、水耗分别下降4%和5%，化学需氧量和二氧化硫排放量分别下降2%和3%；市区空气质量二级以上天数比重力争达到71%。

　　为全面实现上述目标，2009年要着重抓好以下六方面工作：

　　(一)努力扩大内需，保持首都经济平稳较快发展。

　　坚决贯彻落实中央扩大内需的各项政策措施，进一步增强投资和消费对经济增长的拉动作用。

　　促进投资较快增长。一是扩大政府投资。2009年市政府固定资产投资计划安排305亿元，重点向以轨道交通为主的基础设施和促进产业发展方面倾斜，向以改善民生为核心的公共服务领域倾斜，向新城、南城、西南部地区、生态涵养发展区和新农村建设倾斜。二是促进重大项目引进和落地。积极争取中央投资项目落户北京，着力吸引符合首都功能定位的重大项目，发挥“绿色审批通道”作用，抓好征地拆迁、资金配套等前期工作，确保一批重大项目尽早开工建设。三是努力带动社会投资。深化投融资体制改革，完善和落实发展非公经济的政策措施，切实拓宽民间投资渠道和领域。加大贴息和补助力度，发挥财政资金的杠杆作用，引导和扩大社会投资。四是促进房地产投资稳定增长。全面落实国家促进房地产市场健康发展的政策措施，在抓好保障住房建设的同时，着力调整住房供应结构，鼓励居民自住型、改善型住房消费，加快发展存量房市场和住房租赁市场，加强市场监测、分析和信息发布，促进首都房地产业健康稳定发展。五是加强政府投资监管。加大政府投资项目和资金统筹力度，规范投资行为，严格控制成本，确保工程质量，提高投资效益。六是认真做好财税工作。依法加强税收征管，规范非税收入管理。优化财政支出结构，严格控制一般性支出，整合资金，集中力量保增长、保民生、保稳定。通过落实各方面工作措施，力争实现全社会固定资产投资增长15%以上。

　　着力扩大消费。社会消费品零售额预期增长13%左右。主要促进措施，一是增强居民消费能力。切实增加城乡居民收入，健全工资增长机制。落实好就业、社保、就医等改善民生的各项措施，加大对困难群体保障力度，改善居民消费预期。二是推动消费结构升级。积极促进住房消费，合理引导汽车消费，进一步提高文化体育、健康休闲等消费份额，发展电子商务，推动特色街区、老字号企业改造升级。优化信用消费环境，进一步提高信贷消费比重。三是努力扩大外来消费。全面落实推进旅游产业发展意见，综合利用奥运资源，加大旅游促销力度，努力稳定国际旅游，大力发展国内旅游，积极承办大型国际展会，促进旅游会展业发展壮大。四是繁荣农村消费市场。提高农村零售连锁便民店、农业生产资料连锁超市、郊区“一乡一集市”的覆盖面，积极推动“工业品下乡”。支持新城商业发展，打造区域商业中心。五是落实扩大内需的专项工作。采购1000辆节能环保公交车，对更新淘汰黄标车给予补贴，支持组建“绿色车队”，提前完成储备粮增储任务。同时，办好第七届中国花卉博览会，做好经济普查工作。

　　加大对企业的扶持力度。全面落实国家各项相关政策，努力帮助企业特别是中小企业克服困难。扩大中小企业创业投资引导基金、风险投资基金、股权投资基金、中小企业信用再担保资金规模，设立农业担保机构、搭建高端制造业投融资服务平台，为符合条件的企业提供贷款担保服务。鼓励金融机构开展金融创新，增加对中小企业的贷款，积极解决中小企业融资难问题。支持中小企业转型升级，鼓励调整产品结构、进行技术改造和产品创新。降低失业、工伤、农民工大病医疗等社会保险缴费比例，调整基本医疗保险缴费周期，减轻企业和职工负担。加强调查研究，及时了解企业生产经营情况，帮助解决实际问题。

　　加强市场监管。继续推进企业信用信息系统建设，促进部门间信息共享，切实做好标准化工作，全面加强商品质量监管。加大专项整治力度，严厉打击各种违法生产、制假售假行为，整顿和规范市场秩序。及时发布消费警示信息，认真处理消费者投诉。完善食品、药品安全监管网络，加强生产流通全过程监管，实行严格的食品安全质量追溯制度、召回制度、市场准入和退出制度。深入开展食品药品安全大检查，着力加强高风险食品、药品和医疗器械的市场抽验工作，让群众吃得安全、用得放心。加大监测预警和调节力度，促进经济平稳运行。加强价格监管，切实做好日常价格监测和市场调查工作，坚决制止各种不正当价格行为。

　　(二)深入推进产业结构调整，促进经济发展方式转变。

　　坚持把保增长、扩内需、调结构有机结合起来，提高自主创新能力，加快经济结构战略性调整，进一步增强发展的协调性和可持续性。

　　着力推进自主创新。大力推进中关村国家自主创新示范区建设，扩大代办股份转让试点，加强信用担保体系建设，积极发展天使投资和创业投资，推动发行高新技术企业集合债券，促进技术与资本有效结合，不断完善园区创新环境。实施高端领军人才聚集工程，办好海外学人中心，支持企业面向全球引进高端人才。鼓励企业加大研发投入，开展多种形式的产学研合作，重点支持能源、环保、新材料、电子信息等领域共性技术和关键技术研发，促进重点行业的技术改造，带动相关产业发展。制定贯彻国家知识产权战略纲要的实施意见，切实保护知识产权，发展以专利、版权为核心的知识产权产业。深化科研院所改革，进一步整合首都科技资源，加强公益性科技条件平台建设，支持各类创新联盟发展。做好协调服务、配套落实工作，推动国家6个重大科技基础设施和10个重大科技专项建设，同步推进本市18个重大科技专项，支持中央企业在京设立研发机构。推动中国技术交易所建设，促进科技成果转化。实行自主创新技术及产品政府首购、订购制度，集中启动一批产业化项目，增强科技创新支撑首都发展的能力。

　　大力发展现代服务业。围绕建设服务之都，进一步发展壮大生产性服务业和文化创意产业，着力提升发展质量和水平。加快推进金融街扩展和丽泽金融商务区、中央商务区、金融后台服务区建设，积极发展石油、林权、环境等要素市场，大力吸引国内外各类金融机构。继续完善支持政策，加快发展信息、科技、商务服务业和现代物流业。积极培育文化创意产业集群，着力建设一批主业突出、各具特色的产业园区，巩固提升文化创意产业支柱地位。健全体制，强化集聚引领作用，推动高端产业功能区协调发展。设立服务业发展引导资金，完善促进服务业发展的政策体系。实施“北京服务”品牌工程，全力做好中国国际服务交易会筹备工作，着力提升服务业综合竞争能力。

　　积极发展高端制造业。以完善高端产业链为目标，全力推进一批重点项目建设，提升现代制造业发展水平。重点培育集成电路、液晶显示器、移动通讯、计算机及网络产品和数字电视五大产业链，努力扩大生物工程和新医药产业规模，促进高技术制造业快速发展。量身打造个性化服务政策，积极引进高端制造业重大项目，重点发展研发设计、品牌建设和市场营销等高端环节。继续淘汰高污染、高耗能、高耗水的制造业。优化空间布局，推进数字电视、汽车零部件、光机电一体化等一批特色产业基地建设。

　　继续抓好节能减排。严格执行国家环保、节能标准，完善符合首都功能的指标体系、监测体系和考核体系。突出抓好工业、交通、建筑三大领域节能，完成30家市级机关办公用房和800万平方米大型公建节能改造。建立餐饮、娱乐等行业节能规范与标准。制定实施促进节能减排的政策措施，大力支持太阳能、风能、地热、生物质能等新能源开发利用，推进混合动力、氢燃料等替代能源产业化。加快节能技术与产品应用，推广1000万只高效照明产品。建立政府引导、企业为主的投入机制，积极引导中小企业进入节能环保领域创业发展。扩大循环经济试点，搞好资源回收利用重点项目，提高资源再利用水平。深入开展全民节能行动，推行节约环保的生产方式、生活方式和消费模式。

　　(三)加快农村改革发展步伐，统筹城乡、区域协调发展。

　　进一步强化惠农政策，加大投入力度，推进改革创新，促进农村经济稳定发展、农民持续增收，加快形成城乡经济社会发展一体化新格局。

　　加强农村各项制度建设。坚持和完善以家庭承包经营为基础、统分结合的双层经营体制，保持现有土地承包关系稳定和长久不变。建立基本农田保护补偿机制，研究制定促进农户土地承包经营权流转支持政策，搭建农地流转服务平台。尽快制定现状集体建设用地和宅基地确权发证管理办法，探索农户宅基地用益物权保障和节约集约利用的具体途径。继续开展征地多元化补偿安置试点，健全新型农民培养长效机制。完善促进农民专业合作社发展的政策，深入推进农村集体经济产权制度改革，抓好集体林权制度改革。完善农业支持保护制度，健全生态环境补偿机制，对主要特色农作物实施补贴，提高种粮补贴和生态作物补贴标准。建立低收入农户增收帮扶机制。加大农村金融政策支持力度，搭建农业投融资平台，启动农村金融综合改革试验区建设，探索建立支农贷款风险补偿机制和农村信用建设鼓励机制，支持发展村镇银行、农村资金互助社、小额贷款公司，研究建立农业再保险机制。

　　繁荣发展农村经济。坚持生态理念，加快推动农村经济发展方式转变。大力开发农业多种功能，设立奖励资金，支持发展籽种农业、循环农业、休闲农业和科技农业，鼓励探索现代农业新型业态，抓好“菜篮子”工程，新增设施农业4万亩。健全农业科技支撑和社会化服务体系，加强仓储流通设施建设，支持鲜活农产品冷链物流系统建设改造。全面推进农业标准化生产，强化农产品质量安全认证。研究制定农村产业扶持政策，做强农产品加工业，提升乡村旅游业。坚持以业兴镇、以业聚人，加快小城镇建设，促进农村二三产业集聚发展。

　　加快农村基础设施建设。编制农业基础设施建设规划，加强农村水利基本建设，重点解决设施农业高效节水问题，鼓励农民建设小型农村水务设施。完善农村路网体系和公交系统，完成乡道村道大修400公里。大力推进电力、通讯、广播电视系统升级，建成3500个行政村邮站，实现村村通邮。抓紧做好村庄规划编制工作，继续实施新农村“五项基础设施”和“三起来”工程。抓好农村饮用水工程建设，提高垃圾收运处理和污水处理水平，建立健全农村公共设施运行维护体系和管护制度。

　　提高区域统筹发展水平。扎实有序推进新城建设，以交通、能源、环境和公共服务设施为重点，高标准规划、高起点建设、高水平配套，促进产业、功能与人口协调发展。加快南城、西南部地区发展步伐，进一步完善倾斜政策，加强基础设施和产业功能区建设，促进重要功能项目、重大产业项目转移落户。着力抓好城乡结合部地区建设，落实、集成和创新政策，培育发展优势产业，完善基础设施和公共服务设施，逐步实施城市化管理，使之成为城乡一体化发展的示范区。加大资金投入和政策扶持力度，做好5000名山区农民搬迁工作，大力发展沟域经济和休闲旅游等特色产业，积极推动产业转型地区发展替代产业，探索培育浅山区低碳高端产业，促进生态涵养发展区协调发展。

　　(四)切实加强城乡建设，不断增强可持续发展能力。

　　加快生态文明和宜居城市建设，促进经济社会发展与人口资源环境相协调。

　　坚持不懈治理交通拥堵。加强组织协调，开通试运营地铁4号线，加快亦庄线、大兴线等线路建设，实现6条地铁规划线路开工。通过技术改造和设施完善，提高现有轨道交通的运营能力。建成西六环和京承高速三期，开工建设广渠路二期和蒲黄榆路，积极推进宋家庄、四惠交通枢纽建设，继续实施道路微循环系统改造，打通一批断头路。进一步调整优化公交网络，提高服务质量。支持地下停车、立体停车设施建设和使用，整顿停车秩序，出台差别化停车价格政策，减少占道停车，改善步行、自行车出行环境，完善实施交通限行措施，引导居民合理选择出行方式。建立客运交通指挥调度和服务信息系统、停车诱导系统，大力发展智能交通。

　　增强资源能源保障能力。搞好城市热电中心和后备热源建设，启动市区大型燃煤锅炉清洁能源改扩建。实施城市电网配网改造。积极推进陕京输气项目建设，完成六环路天然气管线工程，形成完善的输配主干网。继续实施一批新能源工程，新增热泵供热面积500万平方米。加快南水北调市内配套工程建设，加大城市供水管网和雨污管线改造力度，确保城乡供排水安全。

　　全力推进环境建设。坚持奥运环境建设标准，延续奥运工作机制，全面实施《首都环境建设规划纲要》。落实第十五阶段控制大气污染各项措施，进一步改善空气质量。按照减量化、资源化和无害化原则，制定垃圾收费政策及标准，积极推进生活垃圾分类收集、运输和处理，垃圾资源化率达到37%。加快垃圾焚烧厂和综合处理设施建设，在餐饮企业相对集中街区开展餐厨垃圾资源化处理试点。建设清河再生水厂二期，推进高碑店、卢沟桥污水处理厂升级改造，市区和郊区污水处理率分别达到94%和50%，扩大城乡再生水利用规模。实施长安街大修和沿线景观建设，完成300个老旧小区、150条胡同整治，加大城乡结合部、历史文化保护区、公园、风景名胜区环境整治力度，进一步提升市容环境水平。

　　加大生态建设力度。加快推进京津风沙源治理，启动山区低效生态林改造工程，实施5万亩水源保护林项目，继续开展生态清洁小流域治理和废弃矿山生态修复。建成10个环城郊野公园，加快建设11个新城滨河森林公园，新建改建20处城市公园绿地，提升100条道路绿化水平，开展市区露天停车场绿化试点。完成永定河生态环境综合整治主体工程，加大北运河流域水系综合治理力度，切实抓好莲花河、丰草河等河道治理。深入开展生态区县、环境优美乡镇和生态村创建活动，提高全社会生态环保意识。

　　提高城市服务管理水平。运用奥运成果，推进城市服务管理模式创新。进一步发挥万米网格城市管理信息平台作用，促进企业和社会公众参与，建设城市服务管理长效机制。推动城管日常执法精细化，规范执法行为，维护好城市环境秩序。完善物业管理政策，加快推行物业标准化服务，健全物业纠纷调解机制。深入研究调控人口规模、统筹解决人口问题的途径和举措，创新流动人口服务与管理方式，探索建立人口调控目标责任制。做好流动人口和市内人户分离人员的计划生育工作，稳定低生育水平，提高人口素质。

　　(五)大力发展以改善民生为重点的社会事业，促进社会和谐。

　　进一步加大公共财政投入，继续实施“五无”工程，办好直接关系群众生活的57件重要实事，促进基本公共服务均等化，使民生工作继续走在全国前列。

　　努力稳定就业局势。落实国家相关政策措施，鼓励和引导高校毕业生到城乡基层、中小企业和非公有制企业就业，做好就业指导、创业服务和对困难毕业生的援助工作。加大对就业困难群体、困难地区和农村转移劳动力的帮扶力度，促进6万名农村劳动力转移就业，实现“零就业家庭”至少一人就业。落实和完善创业政策，进一步优化创业环境。建立城乡一体的就业管理制度，在岗位补贴、社会保险补贴、职业技能和创业培训补贴等方面实现城乡同等待遇。以乡镇和村级就业服务机构建设为重点，健全公共就业服务体系。认真贯彻落实劳动合同法，完善解决农民工工资拖欠问题的长效机制。加强失业预警和劳动关系动态监控，实行劳动用工备案制度，大力推行集体合同和工资集体协商，妥善解决劳动争议，维护劳动关系和谐稳定。

　　进一步提升城乡社会保障水平。统一城乡居民养老保险制度，确保现有制度有效衔接。实施城镇无医疗保障老年人和灵活就业人员门诊报销政策，实行企业退休人员医疗费用报销与单位缴费脱钩。提高企业退休人员基本养老保险、城乡最低生活保障、失业保险、工伤保险待遇标准，提高农村五保供养和各类优抚补助水平，提高职工丧葬补助和取暖补贴。实行农村低保对象分类救助，健全医疗救助政策，使各项社会救助政策惠及城乡低收入家庭。完善养老服务补贴政策，大力发展居家养老服务，支持社会力量兴办城乡养老设施，新增养老床位1.5万张。加强慈善文化、制度和组织建设。

　　推进首都教育现代化。制定首都教育中长期改革发展规划纲要。保障教育投入依法增长，新增经费重点投向农村地区、薄弱学校。支持200所小学标准化建设，全面改善农村寄宿制学校生活条件，修缮改造300所农村中小学教师集体宿舍。落实城乡免费义务教育的各项政策，加大对困难家庭学生教育资助力度，提高特殊教育学校学生人均公用经费标准。扩大公办学校接收外来务工人员子女接受义务教育比例，规范打工子弟学校办学和管理。落实义务教育学校实施绩效工资的指导意见，确保教师平均工资不低于公务员平均工资水平。做好教师培训工作，探索优质师资合理流动、均等配置机制，继续完善招生制度，规范学校收支，坚决治理捐资助学与学生入学挂钩，推动义务教育均衡发展。加强和改进未成年人思想道德建设，做好学校体育和健康工作，全面实施素质教育。理顺幼儿园办学体制，加快农村幼儿园建设。促进职业教育特色发展，重点建设70所中职学校，开展中高职教育衔接试验。优化高等教育专业结构，推进重点学科建设，重视大学生思想政治教育和心理健康辅导。培育学习型组织，完善市民终身学习服务体系。

　　加快医疗卫生事业发展。以公共卫生、农村和社区卫生为重点，完善医疗卫生服务体系。继续抓好重大疾病防控工作，实施全民健康行动计划，提高群众健康水平。积极培养引进社区卫生人员，健全社区首诊和双向转诊制，扩大社区基本药品范围。全面加强农村卫生体系建设，落实远郊区县区域医疗中心建设计划，强化乡镇卫生院服务功能，规划建设一批村级卫生室和健康工作室，进一步提高新农合医疗保障水平。强化医疗机构全行业监管，开展单病种医药费用总额预付制试点，规范药品集中招标采购制度，努力减轻群众医药费用负担。

　　促进首都文化繁荣发展。按照中央要求，认真做好新中国成立60周年庆祝活动有关工作。以“人文北京、文明行动”为载体，广泛开展精神文明创建活动。深入实施公民道德工程，推进和谐文化建设，培育文明社会风尚。完善基层公共文化服务体系，推进文化信息资源共享工程，完成所有行政村文化中心、数字电影放映厅建设任务，在全市317个街道、乡镇设立图书服务站点。加大政府补贴，降低演出票价，增加演出场次，组织好面向社区和农村的各类公益文化演出活动。创新文化产品和传播方式，鼓励创造优秀文艺作品，繁荣高端文化演出市场。继续实施文物建筑抢险修缮工程，增加博物馆、纪念馆等公共文化场所向社会免费开放的数量。积极申报非物质文化遗产生态保护区，加大非物质文化遗产的保护和开发力度。支持新闻出版、广播影视、文学艺术和哲学社会科学加快发展，加强互联网等新兴媒体的建设和管理。创新科普工作机制，增强科普实效性。深入开展群众体育活动，支持群众体育组织发展，探索推进竞技体育体制改革，积极培育大型品牌体育赛事，发展壮大体育产业。

　　积极解决群众住房困难。加大住房保障力度，开工建设和收购廉租房、经济适用房、政策性租赁房、限价商品房850万平方米，竣工200万平方米。统筹旧城风貌保护、危旧房改造和人口疏解，完成旧城历史文化保护区2万户居民住房修缮整治，同步整治街巷胡同、完善市政基础设施。全面启动门头沟、通州、丰台“三区三片”棚户区改造试点。推进农村住宅增温节能改造，为农村5500户特困家庭、1000户优抚家庭翻建维修住房。实施5万户平房“煤改电”、20万户热网改造及10万户老楼通气工程，改善居民居住条件。

　　切实加强社会建设。落实社会建设实施纲要，深化社会管理体制改革，推进和谐社会共建共享。完善购买社工岗位、“枢纽型”社会组织管理、社区居民居家养老等政策，不断扩大政府公共服务供给。将社区公共服务设施纳入城市基础设施建设规划，加快城市社区服务站规范化建设，设立200个农村社区服务站。建立专项资金，搭建服务平台，加强社会组织建设、管理与服务。建立健全社会工作者培养、评价、使用、激励机制，进一步完善社会志愿服务的组织体系和工作机制，努力促进社会工作经常化、制度化、规范化。扩大村民自治、社区居民自治，深化村务公开、厂务公开，依法组织第七届社区居委会选举工作。进一步落实党的民族政策，加强基层民族工作，加大对民族乡村发展的扶持力度。全面贯彻党的宗教工作基本方针，认真做好宗教工作。切实做好侨务工作，扩大京台交流与合作。支持工会、共青团和妇联等群众组织开展工作。进一步推动老龄事业发展，切实保障妇女和未成年人权益，继续做好残疾人就业、无障碍设施建设等工作。加强国防后备力量建设，广泛开展双拥共建和国防教育，完善优抚政策，做好军队干部转业安置、退役士兵教育培训和就业服务，增强军政军民团结。

　　全力建设“平安北京”。巩固平安奥运成果，高标准做好安全稳定工作，实现“大事不出、小事减少、管理严格、秩序良好”的目标。全面开展城市风险评估控制及安全隐患排查整改，强化风险与隐患的动态监测。完善突发公共事件总体应急预案，健全基层应急管理体系和首都区域协作联动机制，提升城市应对突发事件能力。深入开展安全生产治理整顿，强化行业和属地监管，建立信息管理平台，全力压减各类事故。健全领导干部接访、重大决策信访风险评估、信访排查督查责任落实机制，重视解决初信初访和重信重访案件，积极防范、妥善处置各类群体性事件。完善公益法律服务体系，扎实做好社区矫正和帮教安置工作，不断提高人民调解水平。加强社会治安综合治理，巩固、规范群防群治队伍，保障平安建设经费，深化精确打击工作机制和多元化治安整治模式，提高科技防范水平，做好反恐防暴工作，努力把北京建成城市秩序最好、群众安全感最高的和谐社会首善之区。

　　(六)着力推进改革攻坚，全方位扩大对内对外开放。

　　抓住有利时机，着眼于建立健全充满活力、富有效率、更加开放、有利于科学发展的体制机制，不断加大改革力度，提升对内对外开放水平。

　　加快重点领域和关键环节改革。深化财政管理体制改革，重点落实市与区县分税制财政管理体制改革方案，提高区县、乡镇财政保障能力。认真贯彻《企业国有资产法》，完善国有资产监管体系。发挥国有资本经营管理中心作用，推动国有大型企业战略并购重组，加快培育千亿元级、具有核心竞争力的大企业集团。适时推进资源产品价格和环境收费改革，促进资源能源节约利用。认真做好增值税、燃油税改革的相关工作。研究制定医疗卫生体制改革方案，探索政府主导与发挥市场作用相结合的医疗卫生服务提供机制，推动公立医院分类改革，鼓励社会资本举办医疗卫生机构。积极申报城市管理综合配套改革试验区，推进城乡结合部地区综合配套改革试点。

　　努力提高开放型经济发展水平。树立全球战略眼光，充分发挥奥运效应，积极利用两个市场、两种资源。完善总部经济发展促进政策，吸引国内外大企业在京设立总部机构。进一步加强投资促进工作，鼓励外资投向现代服务业、高技术产业、节能环保和研发创新等重点领域，提高利用外资水平。完善管理机构和运营机制，加快天竺综合保税区建设。探索建立与天津口岸直通模式，推动解决出口退税和结售汇问题，为企业出口提供有力帮扶。支持企业引进先进技术，扩大关键零部件进口，增加重要战略物资储备。鼓励有实力的企业稳步实施“走出去”战略。研究制定服务外包政策，促进服务贸易发展。

　　积极推进区域合作和对口支援。发挥科技创新和综合服务等优势，找准北京在京津冀都市圈和环渤海地区的定位，加强与周边省区市的联系沟通，拓展交通、能源、水资源、生态保护、旅游发展、生产基地等方面的联合与协作。坚持走高端产业发展之路，避免低端竞争，实现错位发展、协调发展。落实对口支援框架协议，鼓励、引导社会力量积极参与，高质量做好支援什邡市恢复重建及其他对口支援工作。

　　三、加强政府自身建设

　　深入学习实践科学发展观，以“加快改革、优化服务、提高效率”为主题，着力深化行政管理体制改革，努力使政府职能转变有新突破，科学民主决策、依法行政有新进展，服务能力和行政效率有新提高。

　　第一，全面推进依法行政。坚决执行市人民代表大会及其常委会的决议，坚持重大事项报告制度，自觉接受人大监督。认真听取民主党派、工商联、无党派人士和各人民团体的意见，自觉接受政协民主监督。坚持与人大代表、政协委员联系制度，高度重视并认真办理人大议案、建议和政协建议案、提案。广泛征集群众建议，接受社会公众和新闻舆论监督。深入贯彻国务院全面推进依法行政实施纲要，认真完成提请市人大常委会审议的地方性法规草案，修订、制定一批涉及社会管理、城市运行与可持续发展的政府规章。严格按照法定权限和程序行使权力、履行职责，规范行政行为，不断提高依法行政水平。

　　第二，深化政府机构改革。以转变政府职能为重点，扎实做好市政府机构改革方案的组织实施，加紧完成各部门的“三定”工作。精简规范市政府议事协调机构和临时机构。制定区县政府机构改革指导意见，指导完成区县政府机构改革。制定事业单位分类改革意见，选择部分区县和部门进行试点。认真搞好乡镇机构改革试点工作，积极推进乡镇事业站所改革。

　　第三，切实提高行政效能。深化行政审批制度改革，进一步精简审批事项，简化审批程序，下放审批权限，切实提高行政效率。完善全程办事代理制，优化工作流程，改进“一站式”服务。规范政府采购，提升服务水平。健全政府部门协调配合机制，把协调配合纳入部门绩效考核范围，加强监督检查，坚决杜绝推诿扯皮、办事拖沓的现象。深化政务公开，落实政府信息公开条例，推进权力公开透明运行。加快电子政务建设。出台提高政府机关工作效能的意见，健全行政机关绩效管理体系，加强效能监察和行政问责。

　　第四，加强作风和廉政建设。大力弘扬奥运精神，增强全体公务员的忧患意识和进取勇气，大兴调查研究、真抓实干之风，大兴服务基层、服务群众之风。坚持勤俭办一切事业，继续压缩政府部门经常性项目支出，严格控制会议、差旅、出国、接待等费用。贯彻落实十七届中央纪委三次全会精神，加强对公务员特别是领导干部的教育与监督，严格执行党风廉政建设责任制，努力拓展从源头上防治腐败的工作领域，深入开展廉政风险防范管理工作，扎实推进惩治和预防腐败体系建设。强化对重要领域、关键岗位的监察和审计，坚决纠正各种损害群众利益的不正之风，坚决查处违法违纪案件，切实做到“廉洁、勤政、务实、高效”。

　　各位代表！

　　实现2009年经济社会平稳较快发展，建设“人文北京、科技北京、绿色北京”，任务光荣而艰巨。让我们更加紧密地团结在以胡锦涛同志为总书记的党中央周围，高举中国特色社会主义伟大旗帜，以邓小平理论和“三个代表”重要思想为指导，深入贯彻落实科学发展观，解放思想，知难而上，扎实工作，以建设繁荣、文明、和谐、宜居的首善之区的优异成绩，迎接新中国成立60周年！
